# Evaluation Mechanism of Political Discourse: A Holistic Approach

**DOI:** 10.1007/s10936-023-09988-7

**Published:** 2023-07-24

**Authors:** Jing Sun, Zhenqian Liu

**Affiliations:** 1 School of Foreign Languages and Literature, Shandong University, Shandong Jinan, China; 2 School of Foreign Languages, QiLu University of Technology/School of Foreign Languages and Literature, Shandong University, Jinan, Shandong China

**Keywords:** Evaluative meaning, Evaluative resources, The state of the union address, Political discourse

## Abstract

Taking the economic issue of Trump’s First State of the Union Address (SUA) as original data, the present study examined the evaluation features of political speeches by adopting a holistic approach, which includes both macro and micro dimensions. At the macro level, a series of semantic patterns were identified, with Goal-Achievement and General-Example Patterns being the most prevalent. They predetermine the evaluative tone, giving the surrounding statements evaluative meanings, exhibiting the radiating nature of evaluative meaning; at the micro level, a variety of resources have been identified, both explicit and implicit, lexical and syntactical, attitudinal and gradational, which collaborate to reinforce the subjective evaluation, revealing the holistic characteristic in the realization of evaluative meaning. Throughout the analysis, three evaluative mechanisms have been proposed, which are the coupling of meaning, semantic prosody, and tense switching. They collaborate and promote the subjective evaluation to be established and reinforced in a cumulative, gradient or hybrid pattern. In a narrow sense, the present study has partially revealed Trump’s political discourse feature. Broadly speaking, it contributes to the theoretical development of the appraisal framework by refining existing evaluation systems through a holistic research paradigm, which in turn facilitates accurate interpretation of various types of discourse.

## Introduction

As an important component of Systemic Functional Linguistics, Appraisal Theory (Martin & Rose, 2007; Martin & White, 2005/2008) provides a systematic approach to exploring the interpersonal meaning at the level of discourse semantics (Andrew & David, 2020), which makes up the deficiency of Hallidayan approach in paying too much attention to mood and modal systems. Appraisal Theory centers on the evaluative system, which comprises Attitude, Engagement, and Graduation. Attitude refers to the semantic resources that are used to express subjective evaluations, while Engagement and Graduation engage with the source and amplification of various evaluations (see Martin & White, 2005: 38). Since the emergence of Appraisal Theory, scholars have been working on refining the evaluation system, which is accomplished either within the domain of interpersonal meaning, or at the intersection of the three meta-functions. As a result, the overall evaluation system (Liu, 2007), Attitude (Cheng, 2007, 2010; Dong & Li, 2021; Martin & White, 2005), Engagement (Wang, 2003; Wang & Lu, 2010; Wei & He, 2019; White, 2003; Yuan, 2008), and Graduation (Yue, 2012; Zhang, 2008) have been refined, to varying degrees. At the same time, more evaluation resources have been found, lexically, syntactically (Bednarek, 2008; Hunston, 2003), and phonologically (Guan & Wang, 2006; Zhao & Li, 2012). However, more attention has been given to explicit evaluation, with implicit evaluation being largely ignored (Li & Liu, 2019; Shaw, 2004; Wang, 2012; White, 2006). Implicit evaluation is prevalent in discourses (Hamouda, 2003; Ma, 2009: 18), which is more context-dependent and more difficult to identify.

In addition to theoretical exploration, appraisal theory has also been widely used in various discourse analyses (Macken-Horarik, 2004; Martin, 2004; Butt et al., 2004; Jin, 2009a; Tang, 2010; Achugar et al., 2013; Feng & Qi, 2014; Lin, 2015; Tian, 2015; Jiang, 2020; Wang & Qu, 2020), among which political discourse is of particular interest (Maireder & Ausserhofer, 2012; Al-Saeedi, 2017; Hoffmann, 2018). As a basic type of political discourse, political speech contains rich evaluative meanings, by which the speaker can align the audience to accept his political views (idea, proposal, or stance) or legitimize his actions. Previous studies have been conducted mainly at the micro level, concentrating on the summary of various lexical evaluation resources (Bandhar, 2011; Hu & Chen, 2018; Miao & Yang, 2021; Pang, 2013; Zhu, 2015); studies conducted at the macro discursive structure are limited, let alone qualitative studies that incorporate both micro and macro dimensions.

Evaluation is essentially an intersubjective phenomenon, the fundamental purpose of which is persuasion (Tang, 2006). This is especially true of political speech, which is a form of political persuasion organized through the functional differentiation of discourses (Kramsch, 2020). In political speech, evaluation is a crucial strategy that is employed by a speaker to achieve political persuasion. In a sense, the persuasive power of a political speech largely depends on the speaker’s strategic utilization of evaluative resources to “naturalize” or “neutralize” an “ideal reader” position. As an audience, his ability to properly interpret and evaluate the discourse mainly depends on his capacity to recognize the operating mechanism of evaluation as well as deconstruct the "naturalization" process of discourse. Therefore, an in-depth qualitative analysis that encompasses both micro and macro dimensions is necessary for comprehensively dissecting the constructive process of evaluative meanings. Only in this way, can we clarify how the evaluative meanings are being constructed, with what kinds of evaluative devices: Do they work alone or cooperate? If the latter, how is the cooperation realized? An elaborate investigation of these questions is of great academic value for making the appraisal framework more comprehensive and refined, therefore more instructive and applicable.

**Fig.1:**
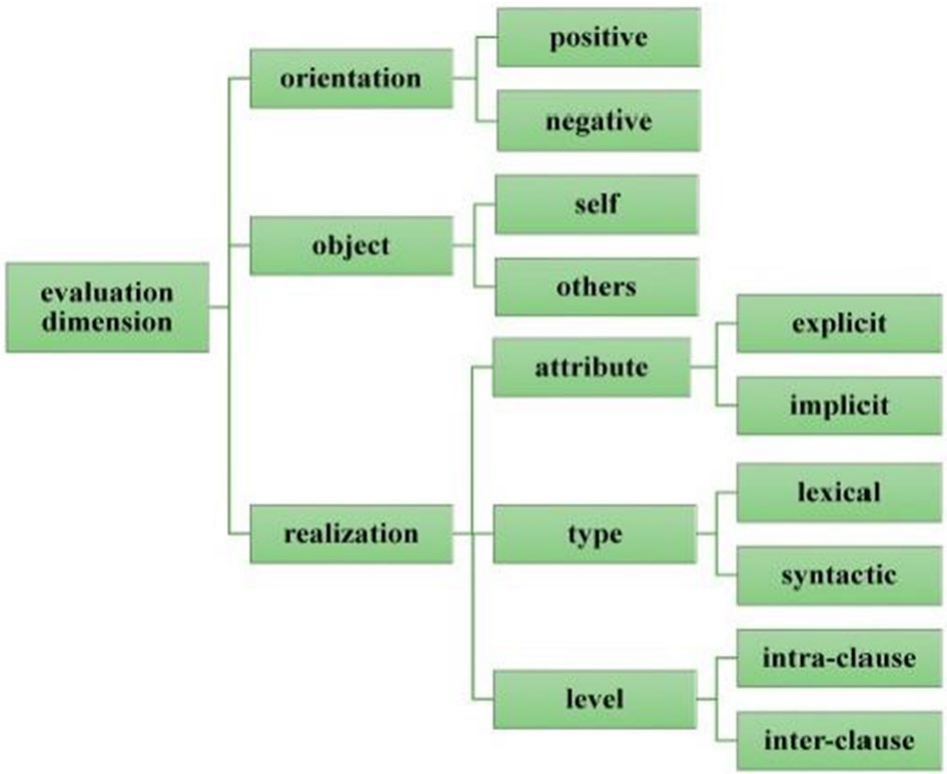
A system of evaluation analysis (after Martin & White, 2005)

With these considerations in mind, the SUA will be taken as data for this study, and the realization mechanism of evaluative meaning will be examined in detail within the framework of Appraisal Theory. Specifically, this study aims to investigate how evaluative meanings are realized and reinforced within a discourse by examining both micro and macro dimensions, with the former mainly involving the orientation of evaluation (positive vs. negative), the object of evaluation (self vs. others), and the realization of evaluative resources (explicit vs. implicit; lexical vs. syntactic; intraclause vs. interclause, etc.) (as shown in Fig. 1), and with the latter concerning the macro semantic pattern of a discourse (as shown in Fig. 3).

**Fig. 2:**
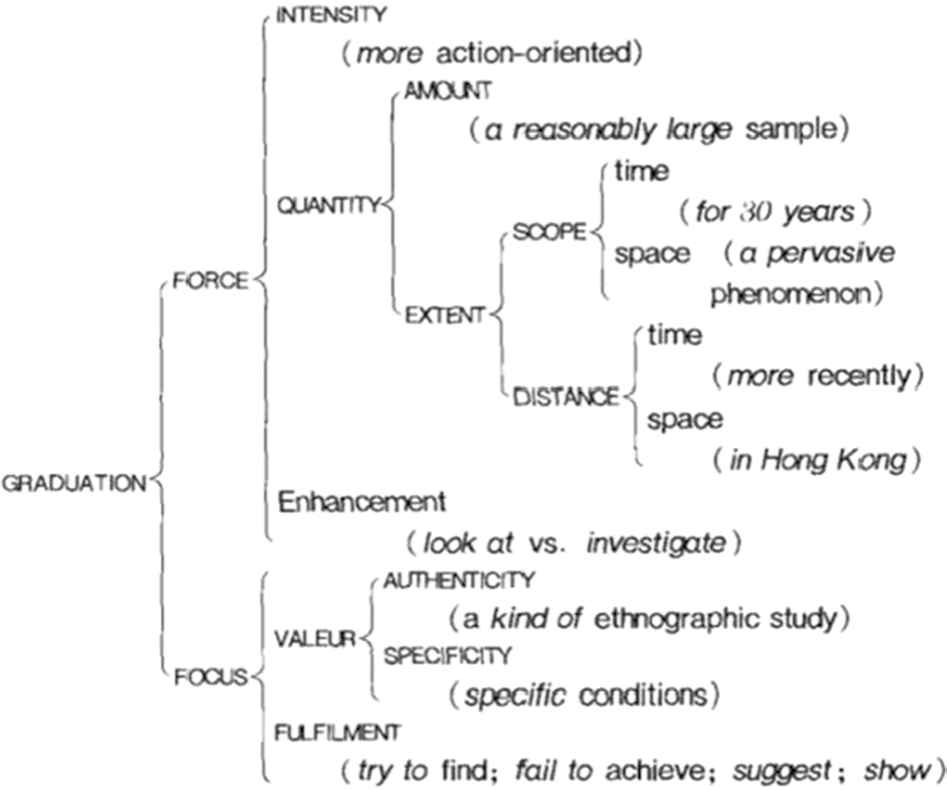
Network of choices in GRADUATION (from Martin & Hood, 2007: 394)

Evaluation is inherently gradable (Martin & White, 2005: 37). Gradation, which is used to regulate the intensity of evaluation, consists of Focus and Force. Considering that Hood and Martin’s (2007: 394) network of GRADUATION (as shown in Fig. 2) is finer and more operative compared with that of Martin and White (2005), this study will be conducted with reference to the former.

This study has six sections, which are arranged as follows. Following the introductory section, the research object––The State of the Union Address (SUA) and Donald Trump as well as his language style––will be emphasized. Then, the research design (including data collection, annotation and research method) will be outlined. Next, an in-depth appraisal analysis will be applied to the data to explore how evaluative meanings are realized in the economic issue of Trump’s first SUA. Following this, the evaluation mechanisms that Trumps prefers to employ will be summarized, which results in the summary of Trump’s evaluation models. The last section concerns the findings and research significance.

## The State of the Union Address (SUA): An Overview

As one of the most significant political speeches for the American President, SUA is an annual formal political report delivered by the President, which includes a comprehensive review of the main jobs that have been accomplished by the current administration in the past year, as well as a prospect of the work plans for the forthcoming year.

The primary audience of SUA is the members of Congress. With advancements in live television technology and other mass media, the audience has expanded beyond the members of Congress to the broader television viewers who can access the speech through various media channels (Lempert & Silverstein, 2012: 151). Along with the expansion of audience coverage, the SUA serves not only as a conduit for work reports but also as a platform to present the president’s political achievements. From this point of view, the SUA is multi-intentional. On the one hand, it provides an important objective window into the current administration's achievements and governing plans; on the other hand, it also serves as a crucial platform for the president to align the public and seek re-election.

## Donald Trump and His Language Style: An Overview

In November 2016, against the polls, projections and estimations, Donald Trump was elected President of the United States. With no previous political experience, he turned from the most discussed and controversial person in modern politics, into the leader of the United States of America in less than a year and a half.

In many ways, Trump represents an exceptional pattern that differentiates him from the typical American politicians. In particular, he is widely renowned for his presidential language, which is believed to deviate from traditional political wordings such as “formal” “solemn” and “politically correct” (Kayam, 2018; Savoy, 2018). Schneider and Eitelmann described Trump’s language as “Trumpolect” (Schneider & Eitelmann, 2020: 7). Trump's political language has been investigated within the domain of political science (Oliver et al., 2016; Lacatus, 2019), sociology (Underberg et al., 2020), communication (McGranahan, 2019) and linguistics (Ahmadian et al., 2017; Denby, 2015; Duran & Lakoff, 2018; Kayam, 2017b; Lakoff, 2016; Nunn, 2016; Ross, 2015). However, previous studies are scattered and fragmented, not sufficient to present the overall characteristics of Trump’s language, which constitutes the point of departure for the present study.

## Research Design

Following the above literature review, the authors selected the economic issue in Trump's first SUA as the original data, investigating how Trump delivers subjective evaluation through seemingly objective government work reports, with what evaluative devices, and in what mechanism.

Genre is a staged, goal-oriented social process (Martin, 1992: 505) that can be divided into different types, such as narrative, descriptive and argumentative. As Zhang noted, “The intervention of any genre difference will bring about changes in language characteristics” (Zhang, 2007). This also applies to evaluative meaning, which is realized differently across genres, following distinct evaluative patterns.

**Fig. 3:**
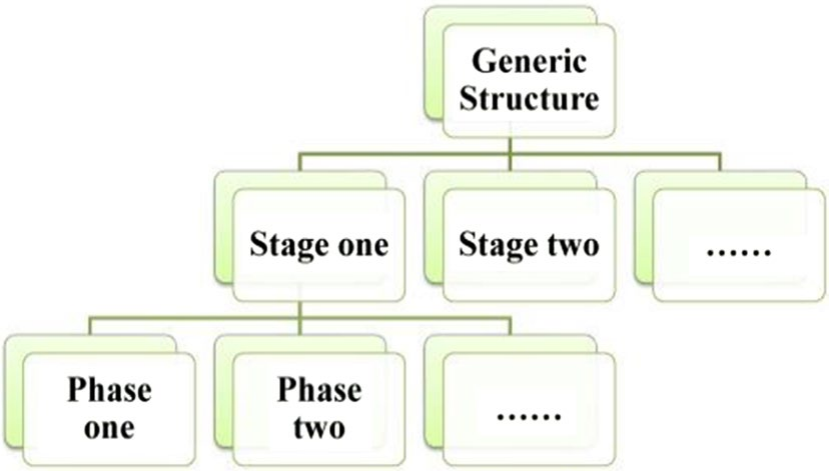
The hierarchy figure of generic structure

Different discourses have different generic structures that are hierarchical and can be divided into various “stages”. Each “stage” is composed of multiple “phases”, which contain one or more pieces of “messages” (Martin & Rose, 2008: 82). In discourse analysis, a discourse is usually divided into several stages, the evaluative meaning of which is realized through distinct pathways at different stages (Cheng, 2010). Taking inspiration from Cheng’s research and guided by Martin’s classification, a refined diagram exhibiting the hierarchical connections of different levels of generic factors was proposed in this study, which is shown in Fig. 3.

**Fig. 4:**
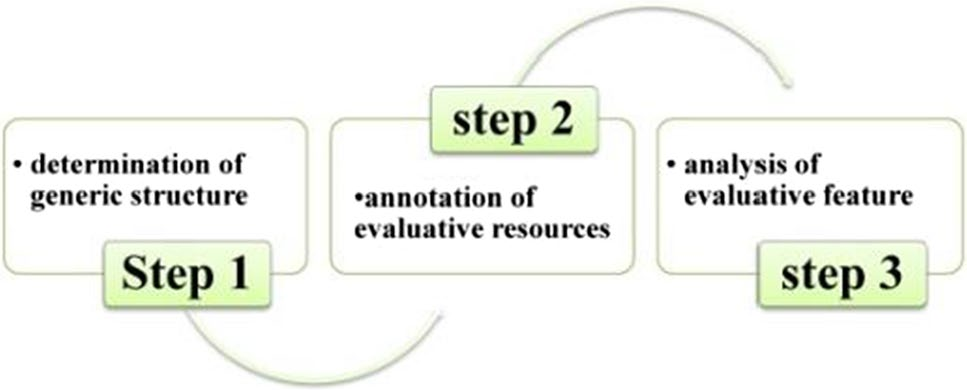
Flow chart for evaluation feature analysis

In summary, a three-step evaluation analysis process that is composed of the determination of generic structure, annotation of evaluation resources, and analysis of evaluation feature was proposed in this study (as shown in Fig. 4). First, we identified and annotated the evaluation resources that appear at different generic stages with reference to Martin and White’s (2005/2008) framework of evaluation analysis. Second, we sorted out and calculated statistics on these evaluation resources, observing how they were distributed, in what patterns, and why.

**Fig. 5:**
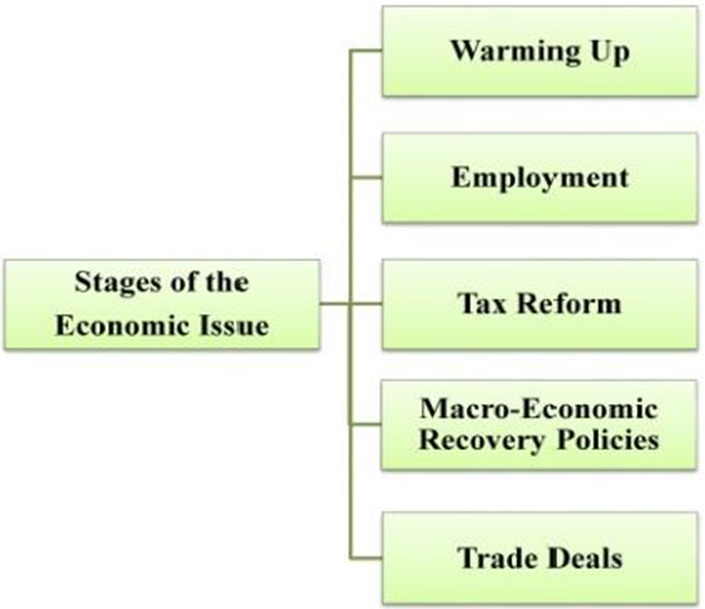
Stages of the economic issue in Trump’s SUA

With regard to this study, the economic issue was subdivided into five generic stages, reflecting different aspects of economic issues, which are: (1) warming-up, (2) employment, (3) tax reform, (4) macroeconomic recovery policies, and (5) trade deals (as shown in Fig. 5). Then, evaluative resources that appear in each discourse were annotated and analyzed.

Systematic evaluation analysis involves simultaneously the orientation of the evaluation, the object of the evaluation, and the realization of the evaluation. Starting at the orientation of the evaluation, evaluation is analyzed according to positive or negative valence, both are of particular interest to our study.

The core of the evaluation system is attitude, which consists of the three basic options of Affect, Judgement and Appreciation (Martin & White, 2005). Affect concerns the semantic resources that are used to construe emotional responses, Judgement concerns resources deployed for construing moral evaluations of behavior, while Appreciation is used to construe the aesthetic quality of semiotic text/processes and natural phenomena.

Drawing on Bakhtin’s (1981) dialogic perspective of language, the engagement system is based on a fundamental distinction between utterances that engage with dialogic alternatives. This distinction is classified as heterogloss and monogloss, respectively (Martin & White, 2005: 98–104). A monogloss proposition does not acknowledge an alternate proposition. The propositions are declared absolutely, which do not explicitly engage in the dialogic alternative. The system of heterogloss, however, acknowledges, to varying degrees, alternate points of view, which is divided into dialogic contraction and dialogic expansion. Dialogic contraction acts to directly reject or challenge alternative propositions, real and/or imagined, and is further categorized as disclaim and proclaim. Dialogic expansion ‘entertains’ or is ‘open’ to dialogic alternatives, real and/or imagined, and is categorized as either entertain or attribute.

Finally, the Graduation system concerns linguistic resources that essentially grade evaluation (Martin & White, 2005: 135–152). It enables a speaker to either up-scale or down-scale the force of his evaluation. Up-scaling essentially increases the speaker’s investment in the evaluation, thus acting to ‘close down’ the dialogic space for the alternative. Down-scaling in contrast decreases the speaker’s investment, thereby distancing himself from the proposition.

With regard to the realization of evaluation, Martin and White (2005) classified them as inscribed or invoked. Under the inscribed category, the evaluation is explicitly presented through a lexical item that carries the value. In contrast, invoked evaluation is realized by the combination of various words, which can invoke Affect, Judgment or Appreciation.

Given the fact that the annotation of evaluative resources is subjective and context-dependent (Fuoli, 2018), the two authors independently made judgments, which were then compared. Where there were differences, there were discussions with reference to the context and the general characteristics of political discourses, and then a final decision was made. To consolidate the reliability of the annotations, the two authors then invited a professor who is familiar with appraisal theory to code those evaluative resources, which is undertaken to establish a third level of interrater reliability in comparison with the two authors' annotations.

In the sections that follow, the study will systematically apply the appraisal framework to the economic issue of Trump’s first SUA to observe exactly how Trump expresses subjective evaluation, with an integrated discussion of Attitude resources (Affect, Judgement, Appreciation), Engagement and Graduation resources being conducted as they are identified across phases in the SUA. For the sake of analysis, each phase of the report will be presented in its coded version (see Table 1 for the appraisal coding system).

**Table 1:** Appraisal coding system

Symbol	Meaning
[]	Attitude coding
+	Positive attitude
−	Negative attitude
**bold**	Inscribed/explicit attitude
underline	Invoked/implicit attitude
box	Engagement
< Angle bracket >	Graduation

## Evaluation Feature in “Warming-Up”

In terms of the generic structure, “Warming-Up” was divided into three stages: (1) “welcome”, (2) generalizing background and (3) elaborating on American heroes, with each consisting of multiple phases. In the following, the evaluation resources employed in each stage and phase will be annotated and analyzed in detail.

### Evaluation Feature in the Second Stage––“Generalizing Background”

Since the stage of “Welcome” is used to greet the audience through expressions such as “*Mr. Speaker*” “*Mr. Vice President*” “*Members of Congress*” “*the First Lady of the United States*” and “*my fellow Americans*”, which can highlight the president's "civility", we will not examine it extensively.

In the second stage, which is composed of three clauses (as shown in < 1 >–< 3 >), Trump stressed the challenges confronting the American people.

Stage two: “Generalizing background”

**Table:** 

Components of semantic pattern	Coded report fragments
Situation	(1) Less than one year has passed since I first stood at this podium, in this majestic chamber, *to speak on behalf of the American People, and to address their concerns, their hopes, and their dreams*[ +]
Method	(2) < That night > , our new Administration had *already taken swift action*[ +]
Result	(3) *A new tide of ****optimism***[ +]* was* < *already* > *sweeping* < *across our land* > [ +]

At the macro level, it follows the Goal-Achievement Pattern (Hoey, 2001: 121), which is composed of Situation, Goal, Method, and Result statements, with the Method and Result statements being necessary components and the Situation being optional. Concerning this stage, Situation, Method and Result are involved.

In Situation (as shown in < 1 >), Trump described how time had flown since his election, which delivers a subjective evaluation of himself through “*to speak on behalf of the American People, and to address their concerns, their hopes, and their dreams*”. This evaluative tone permeated the Method and the Result due to their semantically close links. As a result, the verbal phrase *“taken swift action”* in Method (as shown in Clause < 2 >) is endowed with evaluative meaning, showing praise for the current administration’s high working efficiency. In addition, the marked circumstantial expression *“that night”*, which highlighted the semantic relations between < 1 > and < 2 > , has further strengthened the positive evaluation.

In Result, as is shown in < 3 > , the achievement of “*swift action*” was presented by a maritime metaphor, through which the above positive evaluation was strengthened. As a significant rhetorical strategy, metaphor can provoke evaluation (Martin & White, 2005: 67). Here, as a direct result of the new administration's actions, “*optimism*” was described as a “*tide that is sweeping across the land*”, which reflects Trump's confidence in his governance capabilities, thereby aligning wider audiences. In addition, the coupling of the material process, the present progressive, and the environmental elements of *“already”* and *“across our land”* created a joint effect and strengthened the evaluation.

Throughout the stage of “generalizing background”, a positive evaluation of Trump and his administration is conveyed, mainly through implicit resources (as shown in Table 2), such as ideational meaning, tense switching and maritime metaphor, as well as the graduation resources of EXTENT (Martin & White, 2005: 67). On the whole, the positive evaluation undergoes a process of forming and gradual strengthening, which is in Situation and Method delivered covertly, and further reinforced in Result. From Situation to Method, and then to Result, the positive evaluation is gradually strengthened, which is in line with the “intensification” characteristics of evaluative meaning (Martin & White, 2005: 19).

**Table 2:** Distribution of evaluation resources in stage two: “generalizing background”

Statements	Progressive model	Realization means
Situation	Establishment	Implicit: ideational meaning (IM)
Method	Establishment	Implicit: IM + semantic enhancement relation
Result	Cumulative enhancement	Implicit: IM + present progressive tense + maritime metaphor + SCOPE + DISTANCE

**Fig. 6:**
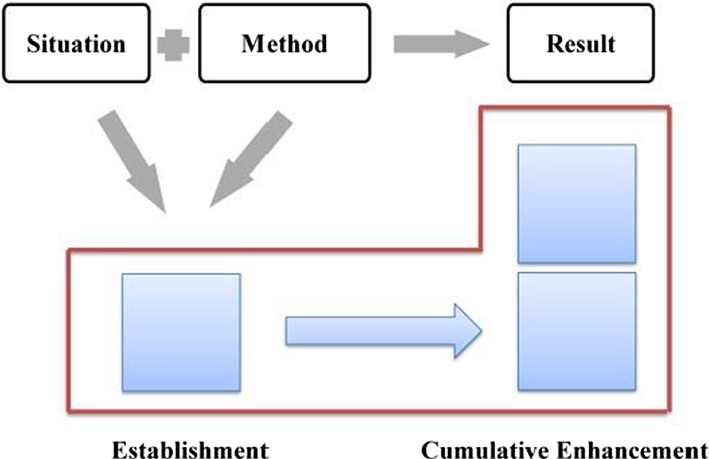
Evaluation feature in “generalizing background”

Compared with explicit evaluation, implicit evaluation leaves more room for negotiation. The audience can choose not to recognize the implied evaluative meaning, in other words, he has the right not to accept the speaker's position or emotional binding. Given the implicit nature of implicit evaluation, this study will take the attribute of evaluative resources as the basic criteria in determining the development characteristics of evaluation, which can be either cumulative or gradient. With regard to the stage of “generalizing background”, the evaluation is established and enhanced “cumulatively” since it is realized mainly through implicit resources (as shown in Fig. 6).

### Evaluation Feature in the Third Stage––“Elaborating American Heroes”

In the stage of “elaborating American heroes”, which follows the pattern of GENERAL-PARTICULAR-CONCLUSIVE GENERAL, Trump praised American heroism through various evaluation resources. Judging from the generic structure, this stage is divided into two phases: (1) outlining brave Americans and (2) elaborating on American heroes, the evaluation feature of which will be analyzed in detail in the following.

#### Evaluation Feature in Phase One of Stage Three: Outlining American Spirit

To begin with, Trump summarizes in general what happened in America over the past year, as depicted in the following (< 4 >).

Phase one of stage three: “Outlining American spirit”

**Table:** 

Components of semantic pattern	Coded report fragments
Generalization	(4a) < Over the last year > , we have made **incredible**[ +] progress and achieved **extraordinary**[ +] success
Specific statement 1	(4b) *We have faced challenges we expected, and others we could never have imagined*[ +]
Specific statement 2	(4c) *We have shared in the heights of victory and the pains of hardship*[ +]
Specific statement 3	(4d) *We have endured floods and fires and storms*[ +]
Conclusive statement	(4e) *But through it all, we have seen the beauty of America’s soul, and the steel in America’s spine*[ +]

**Fig. 7:**
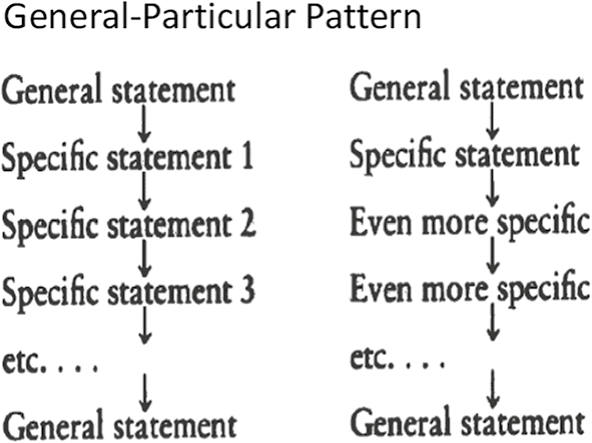
General-Particular Pattern

In terms of semantic structure, this phase conforms to the General-Particular Pattern (Hoey, 1983: 31), which was subdivided into Generalization-Example pattern and Preview-Detail pattern, with the former being further described as *General statement––Specific statement 1––Specific statement 2––Specific statement3……––General statement*, and the latter being shown as *General statement––Specific statement……––Even more specific––Even more specific……––General statement* (as shown in Fig. 7 cf., McCarthy, 1991: 158).

As the Super Theme, this phase follows the Generalization-Example pattern, in which, the evaluative meaning undergoes a process of forming, strengthening and sublimation. Specifically, in < 4a > , the combination of two explicit evaluation resources with high value (*“made incredible progress”*; “*achieved extraordinary success*”) and the inclusive referential of “*we*” deliver overt praise of the American people, which is further enhanced in the Specific Statements (< 4b >–< 4d >). The juxtaposition of “we *have faced challenges*” “we *have shared*” and “we *have endured floods and fires and storms*” has reinforced the praise for the American people’s spirit of sharing joys and sorrows. Moreover, the parallel structure, which begins with “*we have…*”, has intensified this evaluation cumulatively. In < 4e > , which serves as the Conclusive statement, the adversative relation between this clause and the preceding two has further enhanced the positive evaluation. That is, the contrast between “*we have faced/shared/endured*….” and “*But through it all, we have seen the beauty of…*” once again highlighted the praise for the American people. Meanwhile, the architectural metaphor, which labels the spirit of the American people as the “*steel*” of “*America’s spine*” has praised the heroism of the American people, to appeal to a wider audience and win their support.

Throughout phase one, a positive evaluation of the American people has been established and continuously strengthened, which is sequenced as inscribed judgement ^invoked judgement ^ invoked judgement^ invoked judgement^ invoked judgement, presenting an example of intensifying prosody of evaluation. Specifically speaking, Trump praised the American people with explicit evaluation resources, which were intensified by gradation resources, highlighting the coupling of attitudinal and gradational resources (Martin, 2008: 491). Following this, the parallel structure in the Specific Statements further strengthened the subjective evaluation. In the Conclusive statement, this positive evaluation was reinforced to a higher level by the combination of adversative relation and metaphor. Throughout the entire semantic process, various explicit and implicit evaluation resources have coupled, connected, and influenced one another, leading to a continuous strengthening of the evaluative meaning, forming a positive evaluation cohesion (Lemke, 1998).

**Fig. 8:**
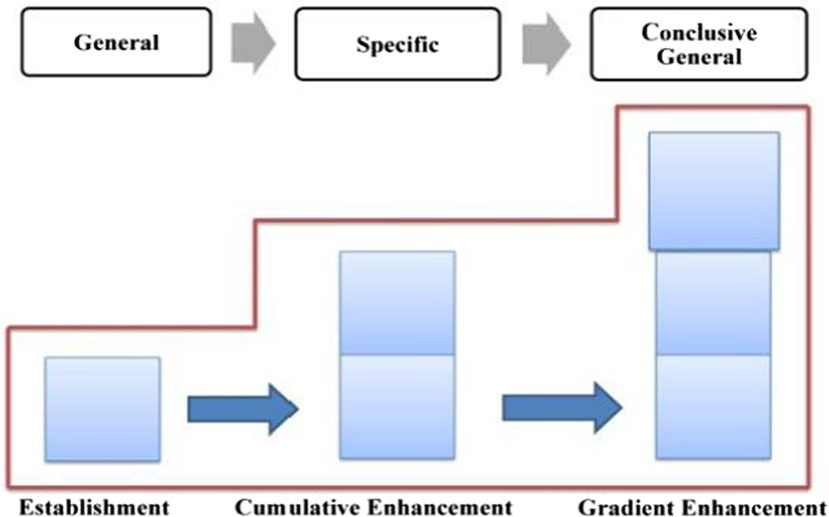
Evaluation feature in “outlining American spirit”–phase one

The distribution of evaluation resources in this phase is shown in Table 3, in which, both explicit and implicit resources are involved, with the former being employed to establish a positive evaluation tone, while the latter to enhance it. In general, Trump’s positive evaluation of the American people goes through a process of forming, cumulative enhancing, and gradient enhancing, which is referred to as “a hybrid pattern” (as shown in Fig. 8).

**Table 3:** Distribution of evaluation resources in phase one of stage three: “outlining American spirit”

Elements	Progressive model	Realization means
General statement	Establishment	Explicit: attitudinal lexes + gradational resources
Specific statements	Cumulative enhancement	Implicit: IM + parallel structure
Conclusive general	Cumulative enhancement	Implicit: semantic shift + metaphor

**Fig. 9:**
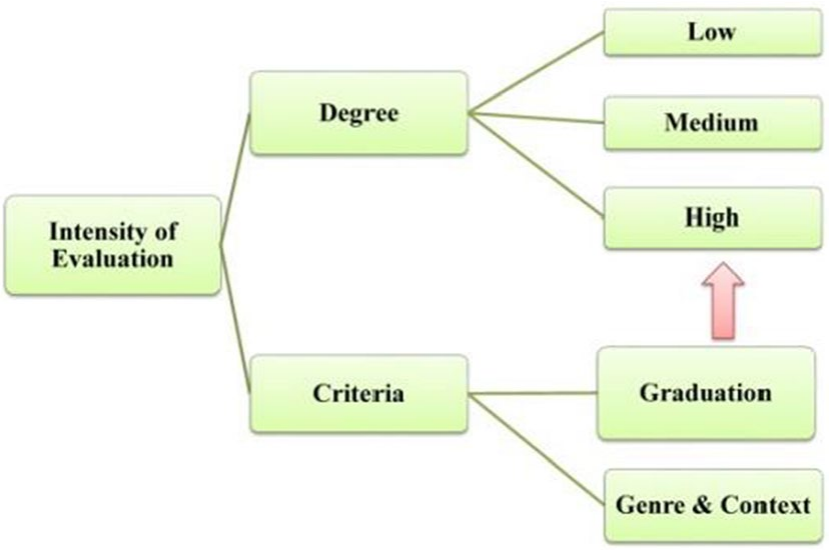
Diagram of evaluation intensity

The intensity of evaluation serves to describe the degree of evaluation, which is subdivided into high, medium, and low. The intensity of valuation is relative and tends to vary across contexts and genres. Evaluation intensity is usually differentiated by gradation resources (Feng & Su, 2021); moreover, the superposition of different evaluation resources can also increase the intensity of evaluation (Cheng, 2015: 2). In general, evaluation with higher intensity is manifested mainly through repetition, sub modification, exclamatory, as well as adjectives of the highest degree. In addition, the employment of various implicit resources, such as parallel structure and metaphor, can also strengthen the intensity of evaluation. The distinction among high, medium, and low intensity of evaluation is also relative and may be dynamic across contexts or generic stages. Overall, the general characteristics of the evaluation intensity are shown in Fig. 9.

#### Evaluation Feature in Phase Two of Stage Three: Elaborating American Heroes

In the second phase, Trump listed in detail the heroic deeds that occurred in the past year, which follows the Generalization-Specific Pattern. In Generalization, Trump used the explicit attitudinal lexis––“heroes” to show his praise of American heroism (as shown in < 5 >), aiming at uniting American people to the greatest extent. The General-Specific relationship between < 5 > and < 6 >–< 7 > promotes the positive evaluation tone to penetrate < 6 > and < 7 > , giving them evaluative meaning, which embodies the radiation function of evaluation (Hood, 2004: 144).

Phase two of stage three: “Elaborating American spirit”

**Table:** 

Components of semantic pattern	Coded report fragments
Generalization	(5) “*Each test has forged new American ****heroes****[* +*] to remind us who we are, and show us what we can be”*[ +]
Specific statement 1	(6) *We saw the volunteers of the “Cajun Navy”, racing to the rescue with their fishing boats to save people in the aftermath of a* < *totally* > ***devastating***[*−*]* hurricane*[ +]*. (7) And we saw strangers shielding from a hail of gunfire on The Las Vegas Strip*[ +]
Specific statement 2	(8) We heard tales of Americans like Coast Guard Petty Officer Ashlee Lepper, who is here tonight in the gallery with Melania. *Ashlee was aboard one of the first helicopters on the scene in Houston during Hurricane Harvey*[ +]. < Through 18 h of wind and rain > , *Ashlee braved live power lines and deep water, to help save more than 40 lives*[ +]. Ashlee, we all Thank you; (9) We heard about Americans like firefighter David Dahlberg. He is here with us too. *David faced down walls of flame to rescue* < *almost* > *60 children trapped at a California summer camp threatened by those devastating wildfires*[ +]
Specific statement 3	(10) With us tonight is one of the **toughest** [ +]people ever to serve in this House, *a guy who took a bullet, almost died, and was back to work three and a half months later: the legend from Louisiana, Congressman Steve Scalise*[ +]. We are < incredibly > **grateful**[ +] for the **heroic**[ +] efforts of the Capitol Police Officers, the Alexandria Police, and the doctors, nurses, and paramedics who saved his life, and the lives of many others in this room

In Specific Statement 1 (< 6 >–< 7 >), Trump portrayed the heroic deed of two ordinary Americans, showing in detail how ordinary Americans responded bravely to devastating and gunfire. On the one hand, the radiation function of evaluation assigns neutral words such as *“racing”* and “*shielding*” with evaluative meaning, which highlights praise of the American people; on the other hand, the semantic contrast between “*rescue*” “*save people*” and *“devastating hurricane”* further enhanced Trump’s high regard for the heroic spirit of American people in the face of natural disasters, which was intensified by the graduation resource of INTENSITY “*totally*”. Similarly, in < 7 > , the semantic contrast between *“a hail of gunfire”* and “*strangers shielding strangers*” also highlighted the praise for the bravery of ordinary Americans.

From an epistemological perspective, American people hold individual experiences in high regard, which they believe to be both authentic and reliable (Zhang & Sun, 2015). In the speech, Trump employed concrete words to describe specific things, thereby intensifying the psychological impression of the audience and appealing to the national trend of empiricist thinking. From a rhetorical perspective, story-telling serves to restore individual experiences with concrete and straightforward expressions, which can evoke empathy in the audience, fostering an intersubjective relationship (Kádár & Zhang, 2019).

Following Specific Statement 1, Trump proceeded to present another anecdote that constitutes Specific Statement 2 and highlighted the courageous actions of an officer and a firefighter (as shown in < 8 >–< 9 >). He described how the officer and the firefighter heroically rescue individuals in the face of natural disasters. Specifically, Trump provided an intricate description of how Coast Guard Petty Officer Ashlee Lippert made efforts to rescue over 40 individuals during Hurricane Harvey. The radiation function of evaluation gives this neutral narration a positive evaluation tone. As a discourse strategy, narration involves more than simply providing an objective depiction of events (Jovi, 2020), but also contains the narrator’s subjective attitude or stance. In this case, the in-depth portrayal of Ashlee’s actions that unfolded during Hurricane Harvey is to evoke the audience’s emotional response, thereby obtaining a positive evaluation of Ashlee. In addition, The QUANTITY expressions of *“the first helicopters”* and *“through 18 h of wind and rain”* further increased the intensity of evaluation.

Following Ashlee’s heroic action, Trump narrated a firefighter named David Dahlberg (as shown in < 9 >). On the one hand, the positive evaluation tone from the Generalization (< 5 >) gives the neutral narration evaluative meaning. On the other hand, the semantic contrast between “*rescue 60 children*” and “*devastating*”, as well as the graduation resource of “*almost*” has intensified the evaluation.

The clause set < 10 > , which is categorized as Specific Statement 3, involves the heroic deed of a congressman. Trump initiated a positive evaluation of Steve through the explicit attitudinal expression of “*the toughest people*”. In addition, a series of material processes were employed to strengthen the evaluation. In particular, the semantic contrast between “*almost died*” and “*was back to work three and a half months*” has largely intensified the evaluation. Besides that, Trump spoke highly of those medical staff who treated Steve and others, with such explicit evaluative resources of high value as “*incredibly grateful”* and *“the heroic efforts*”.

**Fig. 10:**
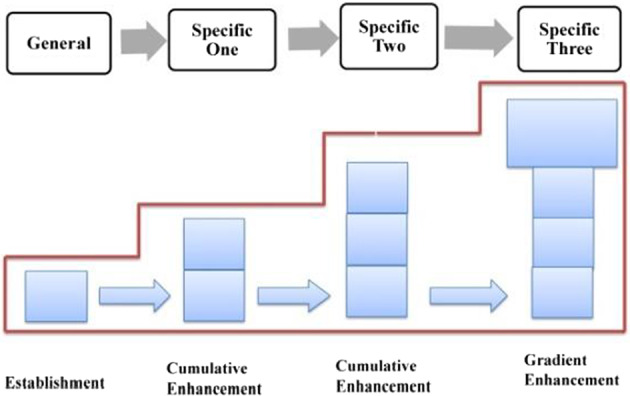
Evaluation feature in “elaborating American heroes”–phase two

In summary, evaluative meanings in Specific Statements 1, 2 and 3 are realized differently, with the former two being realized mainly by implicit resources and the third being realized mainly by explicit resources (as shown in Table 4). In general, the evaluation of this phase develops in a process of establishment ^ cumulative enhancement ^cumulative enhancement ^ gradient enhancement, which is a hybrid pattern (as shown in Fig. 10).

**Table 4:** Distribution of evaluation resources in phase two of stage three: “elaborating American heroes”

Elements	Progressive model	Realization means
General	Establishment	Explicit: attitudinal lexis
Specific 1	Cumulative enhancement	Implicit: radiation function + IM + semantic contrast
Specific 2	Cumulative enhancement	Implicit: radiation function + IM + semantic contrast relationship + story telling
Specific 3	Gradient enhancement	Explicit and implicit: explicit means + radiation function + IM + semantic contrast relationship + story-telling

#### Evaluation Feature in Stage Three: Summary

Based on an extensive analysis of phases one and two, which were classified into the SPECIFIC STATEMENT of the stage of “Elaborating American Heroes”, we have figured out how Trump expressed and enhanced subjective evaluation of the American people, which, in the CONCLUSIVE STATEMENT (as shown in < 11 >), is reinforced once again.

Conclusive GENERAL of phases one and two

**Table:** 

Components of semantic pattern	Coded report fragments
Conclusive statement of phases one and two	(11a) Over the last year, the world has seen what we always knew: *No people on Earth are so ****fearless****, ****daring****, or ****determined***[ +] *as Americans*[ +]*. (11b) If there is a frontier, we cross it. If there is a challenge, we tame it. If there is an opportunity, we seize it*[ +]

To begin with, the combination of attitudinal lexes (“*fearless*”*,* “*determined*”) and the structure (“*no…as…*”) in < 11a > echoes the above positive evaluation. In addition, the rhetorical parallel structure in < 11b > (“*if there*….., *we do*……”) presents Trump’s determination and executive power as the president. The simple present tense, appearing in < 11b > , which projects Trump’s determination to serve the American people, not in the past or the future, but now, further highlights the positive evaluation. To sum up, in the CONCLUSIVE GENERAL, the positive evaluation is continuously reinforced by the coupling of various resources, both explicit and implicit, lexical and syntactic, which forms a mutually reinforcing relationship with the GENERA.

In summary, in the whole stage of “Elaborating American Heroes”, Trump established a positive evaluation of the American people through the coupling of various resources. At the same time, the evaluation tone of the General has penetrated the Specific statements, predetermined its evaluative meaning, and exemplified the radiation function of evaluation. To sum up, the evaluative meaning within a phase is continuously enhanced, presenting a cumulative or hybrid pattern, with the latter being composed of cumulative and gradient enhancements.

## Evaluation Feature in the Employment Issue

Unlike in “warming-up”, where Trump’s evaluation points to American people, the object of evaluation in the issue of employment, which can be divided into the stage of “job growth” and “unemployment” rate, is Trump and his administration.

### Evaluation Feature in Stage One––“Job Growth”

“Job growth” is composed of two clause complexes, as is shown in < 12a > and < 12b > .

Stage one: “job growth”

**Table:** 

Components of semantic pattern	Coded report fragments
Method	(12a) < Since the election > , *we have created 2.4 million ****new***[ +]* jobs, including 200,000 new jobs in manufacturing* < *alone* > [ +]
Result	(12b) *After years and years of wage ****stagnation***[*−*]*, we are* < *finally* > *seeing ****rising*** [ +]*wages*[ +]

On a macro scale, it follows the Goal-Achievement Pattern, with < 12a > being the Method, and < 12b > being the Result. In < 12a > , Trump described the changes that had taken place in the job market since his election. This seemingly objective statement affords a positive evaluation of Trump himself, which is realized by the combination of the verb “*created*” and the specific number “*2.4 million jobs*”. In addition, the nonpredicate structure (“*including 200,000 new jobs in manufacturing alone*”) has reinforced this positive evaluation by extending the main clause semantically, emphasizing the finer achievements that Trump and his administration had accomplished. This positive evaluation undergoes a secondary reinforcement by the graduation resource of “*alone*” at the end of the clause (Hood & Martin, 2010: 390). Moreover, the inclusive referential––“*we*”, which implies a common identity with the audience, was used by Trump to reduce the suspicion of “boasting” by stressing that the achievements are the result of joint efforts of himself, the current administration and the Congressmen, thereby increasing the acceptability of evaluation. This blurring reference can also reduce interpersonal tasks, contributing to strengthening the alliances with actual and potential audiences (Liu & Chang, 2021).

**Fig. 11:**
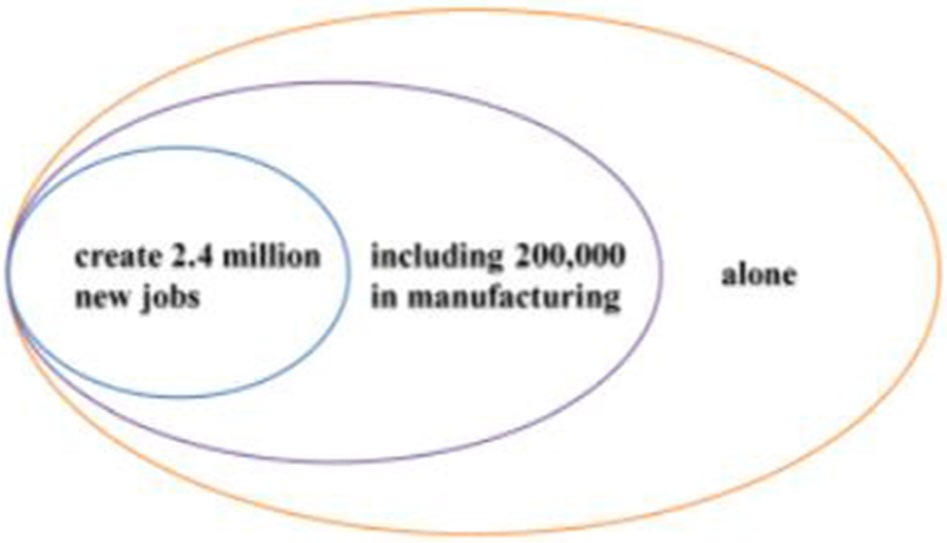
Intensified positive evaluation prosody

In short, from ideational meaning, to verb phrases with a specific number, and graduation resources, Trump’s evaluation of himself and the administration is actively constructed throughout < 12a > , and becomes increasingly intense, thus forming an “enhanced positive evaluation prosody” (as shown in Fig. 11), which aligns with the “saturation” characteristics of evaluative meaning (Martin & White, 2005: 19).

In the Result (as shown in < 12b >), Trump once again stressed the achievement that he and his administration had made through the semantic contrast between “*wage stagnation*” and “*rising wages*”, “*years and years*” and “*finally*”, “*after*” and “*are seeing*”. He simultaneously invoked a negative evaluation of the previous administration as well as a positive evaluation of the present administration, as whatever is “bad” for the previous administration is in effect “good” for the present administration (Andrew & David, 2020). As an evaluation strategy, semantic contrast is based on semantic reasoning rather than “empty talk”, which tends to be more covert yet less open to challenge.

**Fig. 12:**
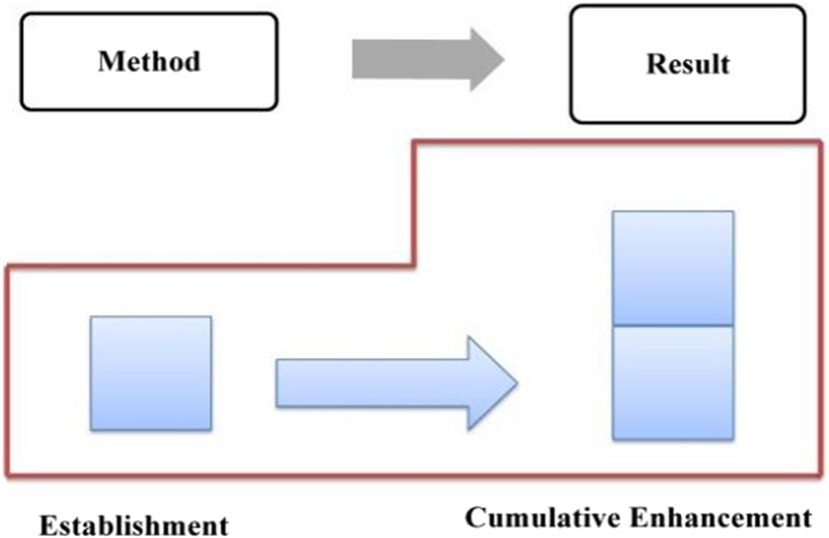
Evaluation feature in job growth

Combining < 12a > and < 12b > , the evaluative meaning is established and reinforced cumulatively, which is realized mainly through implicit resources. The evaluation tone established in Method is further reinforced due to the Method-Result Pattern, revealing the transitivity of evaluative meaning. In Method, the “neutral” ideational meaning affords a positive evaluation of Trump and his administration (Martin & White, 2005: 67), which is further reinforced by the coupling of specific numerals and graduation resources. In Result, the multi-level semantic contrast has created a cumulative force, highlighting the praise for the policy implementers––Trump and his administration. The distribution of evaluation resources throughout the first stage is presented in Table 5, with a cumulative evaluation model being formed (as shown in Fig. 12).

**Table 5:** Distribution of evaluation resources in stage one: “job growth”

Statements	Progressive model	Realization means
Method	Establishment	Implicit: IM + specific number + extension relation + graduation resource of ENHANCEMENT
Result	Cumulative enhancement	Implicit: multi-level semantic contrast relationships

### Evaluation Feature in Stage Two: “Unemployment Rate”

In the second stage, which talks about “the unemployment rate” (as shown in < 13 >), evaluative meanings are realized through various attitudinal resources, accompanied by a series of graduation resources.

Stage two: “unemployment rate”

**Table:** 

Components of semantic pattern	Coded report fragments
General	(13a) *Unemployment claims have hit a 45-year low*[ +], something I am very **proud**[ +] of
Specific 1	(13b) *African-American unemployment stands at the ****lowest***[ +]* rate* < *ever recorded* > [ +];
Specific 2	(13c) *And Hispanic American unemployment has also reached the ****lowest***[ +] *levels* < *in history* > [ +]

As a semantic whole, it follows the General–Example Pattern, with < 13a > serving as General and < 13b > and < 13c > as Specific 1 and Specific 2, respectively. In General, Trump emphasized his work achievements by outlining that American unemployment claims have reached a 45-year low, through which, a positive evaluation of himself and his administration is evoked, for a decrease in unemployment is in line with people’s psychological expectations. In particular, the co-occurrence of “*45-year*” and “*low*” has largely intensified the evaluation by highlighting the great contribution that the current administration has made, aiming at inspiring the audience to make similar evaluations. The addition of the infused process of INTENSITY (*“something I am very proud of”*), which elaborates with the main clause semantically, intensifies once again the positive evaluation. By embedding this process, which is led by “*I*”, Trump closed the possibility for an alternative dialogic position, underlining his self-confidence in economic decision-making, as well as his unwillingness to leave room for audience dissent (Hood & Martin, 2007: 386).

In Specific Statements 1 and 2, as is shown in < 13b > and < 13c > , Trump listed the unemployment rates of African-American and Hispanic-American people, respectively, through which the evaluation was enhanced further. More specifically, the co-occurrence of the *“unemployment rate”* and the inscribed attitudinal lexis with high-value *“lowest”* has reinforced the positive evaluation, which is strengthened by the graduation resources of EXTENT “*ever recorded*” *and* “*in history*”, revealing the “intensifying prosody” characteristics of evaluative meaning (Martin & White, 2005: 24).

**Fig. 13:**
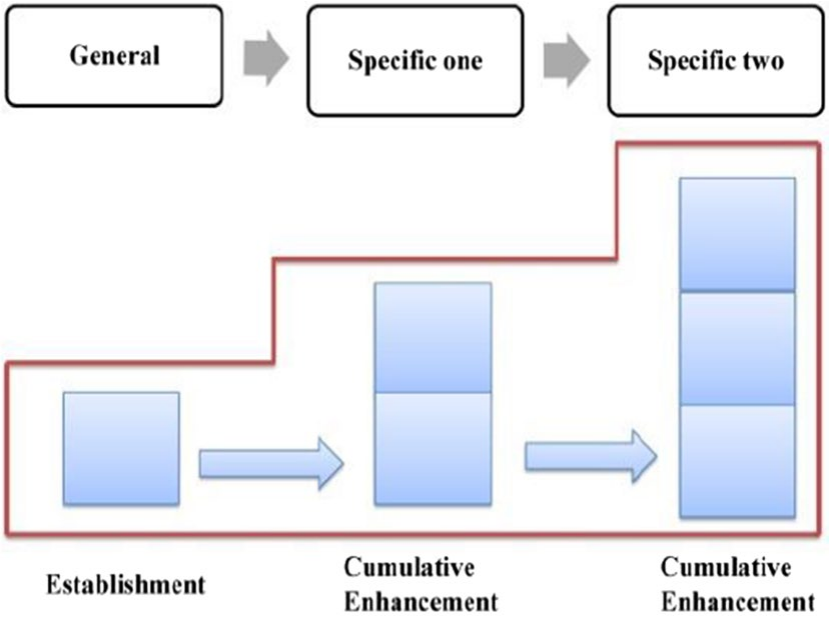
Evaluation feature in unemployment rate

In summary, during stage 2, evaluative meanings are expressed through resources such as ideational meaning, specific numbers, infused processes, and graduation resources (as shown in Table 6), accordingly, the positive evaluation develops in a process of establishment and cumulative enhancement (as shown in Fig. 13).

**Table 6:** Distribution of evaluation resources in stage two: “unemployment rate”

Statement	Progressive model	Realization means
General	Establishment	Implicit: IM + specific number + infused process of INTENSITY
Specific 1	Cumulative enhancement	Implicit: IM + graduation resource of INTENSITY
Specific 2	Cumulative enhancement	Implicit: IM + graduation resource of INTENSITY

## Evaluation Feature in “Tax Reform”

The third topic in the economic issue involves “tax reform”, which focuses on the benefits to America. Judging from the discursive structure, it follows a GENERAL––SPECIFIC Pattern. Within the GENERAL, a Goal-Achievement Pattern is embedded, with < 14 > serving as the Method and < 15 > as the Result, indicating the “overlapping of different patterns within one text or discourse” (Hoey, 2001: 148).

First, in Method (as shown in < 14 >), Trump generalized the tax cuts that were implemented by his administration.

The GENERAL.

**Table:** 

Components of semantic pattern	Coded report fragments
Method	(14) *Just as I promised the American people from this podium 11 months ago, we enacted the ****biggest***[ +]*tax cuts and reforms* < *in American history* > [ +]
Result	(15) *Our massive tax cuts provide ****tremendous***[ +]* relief for the middle class and small businesses*[ +]

Shared knowledge gave the verb phrase “*enacted…tax cuts*” evaluative meaning, which was intensified by the attitudinal lexis “*biggest*” and the graduation resource of EXTENT (“*in American history*”). The coupling of these two resources doubles the force of evaluation. In addition, the subordinate clause led by “as”, which maintains a concession relation with the main clause, has increased the intensity of evaluation, indicating that Trump is a president who keeps his word.

In Result, as is shown in < 15 > , the effect of massive tax cuts was outlined, which provokes a positive evaluation of the implementer––incumbent administration. The employment of the verb phrase “*provide relief”*, which was modified by the explicit lexis (*“tremendous”*), further intensified the evaluation.

**Fig. 14:**
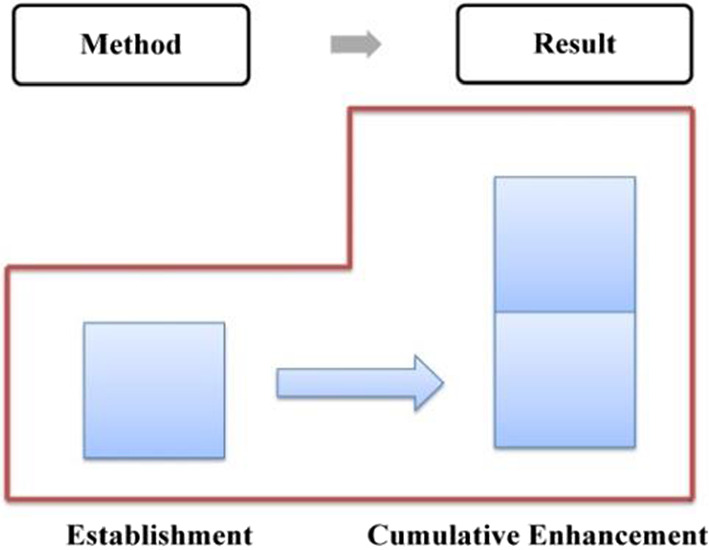
Evaluation feature in the GENERAL

Taking Method and Result into consideration, the evaluative meaning in GENERAL is realized and reinforced by diverse resources, as is exemplified in Table 7. In general, the evaluation develops in a cumulative pattern (as shown in Fig. 14).

**Table 7:** Distribution of evaluation resources in the GENERAL of “tax reform”

Statements	Progressive model	Realization means
Method	Establishment	Implicit: IM + concessive relationship + attitudinal lexis + graduation resource of EXTENT
Result	Cumulative enhancement	Implicit: IM + modifier lexis

### Evaluation Feature in Stage One–– “Tax Cuts for American People” (SPECIFIC One)

In the first SPECIFIC, the benefits that the tax cuts have brought to middle-class and low-income families are elaborated, which will be presented in the following.

- (1) Phase one: tax cuts for the ordinary middle class

SPECIFIC one consists of two phases, with the first phase following the Goal––Achievement––Conclusive Statement Pattern and the second following Goal-Achievement Pattern. In the first stage (as shown in < 16 >), Trump described how his policy has benefited the ordinary middle class. Phase one of SPECIFIC one: “tax cuts for the ordinary middle class”

**Table:** 

Components of semantic pattern	Coded report fragments
Method 1	(16a) *To lower tax rates for ****hardworking**** [* +*]Americans, we nearly doubled the standard deduction* < *for everyone* > [ +]
Result 1	(16b) < *Now* > *, the first $24,000 earned by a married couple is* < *completely* > *tax-free*[ +]
Method 2	(16c) *We also doubled the child tax credit*[ +]
Result 2	(16d) *A typical family of four making $75,000 will see their tax bill reduced by $2,000, slashing their tax bill in half*[ +]
Conclusive statement	(16e) *This April will be the last time you ever file under the ****old***[*−*] *and* < *very*** > *broken***[*−*]* system, and millions of Americans will have more take-home pay starting next month*[ +]

In < 16a > , which serves as Method 1, by highlighting that the actions that the incumbent administration has taken are beneficial for hardworking Americans, a positive evaluation of Trump and his administration is invoked. Specifically, the goal––means relationship between the two clauses gives the verb phrase *“doubled standard deduction*” positive evaluative meaning, which is further enhanced by the EXTENT of *“for everyone*” by emphasizing that the tax cut policy benefits every American citizen. In < 16b > , which serves as Result 1, Trump presented the benefit of their tax reform by stressing that the first earned money for a married couple is tax-free, which flags praise for their tax cut. At the same time, the coupling of the specific number “$24,000”, “*tax-free*” and the EXTENT resource (“*completely*”) produces a superposition effect, presenting the audience with the huge benefits that they will get from the tax cut, through which the positive evaluation is further reinforced.

 < 16c > and < 16d > , which described another tax cut policy––child tax credit––also follow the Goal-Achievement Pattern. In < 16c > , which serves as Method 2, a positive evaluation is activated from the ideational meaning since the verb phrase “*doubled the child tax credit*” is in line with people’s psychological expectations. In Result 2 (as shown in < 16d >), which describes how the child tax credit benefits American people, this positive evaluation is further enhanced through various resources, such as the ideational meaning and the semantic contrast of different specific numbers. In addition, the infused process of AMOUNT (“*slashing their bill in half*”), which keeps an elaboration relation with the main clause, has also invoked positive valuation by showing that the child tax credit can benefit the American people through significantly lowing their bills.

Following the above two groups of Goal-Achievement Patterns, Trump promised that next month would be the last time that American workers file their taxes under a very broken system (as shown in < 16e >), through which, the evaluation meaning is strengthened to a higher level. Here, by criticizing overtly the previous tax system as “*the old and very broken system*”, a positive evaluation of the current administration is invoked, which reveals the deployment of “going negativity”.

“Going negativity” refers to the focus within campaigning processes being placed upon alleged faults and weaknesses of opposition candidates rather than on a candidate’s own personal and policy strengths (Dolezal et al., 2017). Negativity can take different forms, but primarily focuses on “attacking rival parties/candidates and criticizing their policy platforms or personality traits” (Ceron & d’Adda, 2015), or at times a combination of both. When policy is the target of negativity, it tends to be the ideas, proposed programs, and track record of opponents that are in the crosshairs (Andrew & David, 2020). Back into this case, through the use of the explicit attitudinal phrase “*the old and very broken*”, a negative judgment of Obama’s tax system is inscribed, which in turn invokes a positive judgment of Trump’s administration, indicating that “going negativity” can realize a speaker or writer’s subjective evaluation. By denying the previous administration’s policy, Trump intended to give credit to his administration, for the semantic contrast between clauses transforms an inscribed negative evaluation of Obama into an invoked positive evaluation of Trump. Immediately following it, the second clause, which keeps a semantic progression relation with the former (Yang, 2003), further reinforces this positive evaluation by stressing that “*millions of Americans will have more take-home pay starting next month*”, especially the model verb “*will*”, implying that Trump is keeping his word, therefore being credible.

**Fig. 15:**
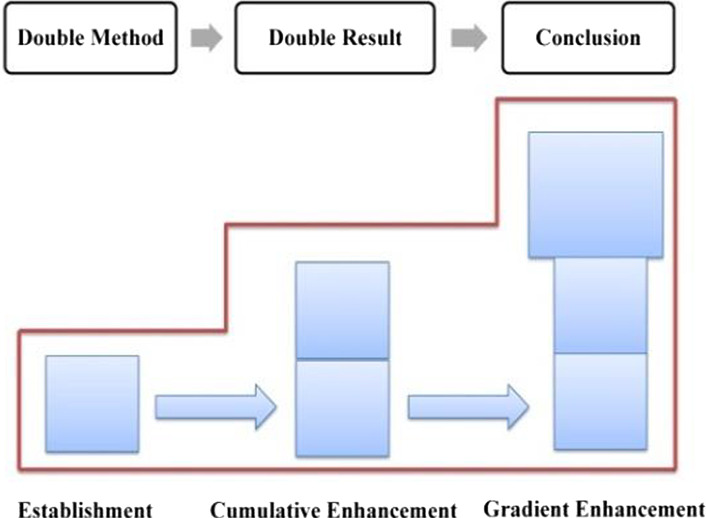
Evaluation feature in tax cuts towards middle class

Throughout the reporting of the tax cuts for the middle class, a positive evaluation of Trump and his administration has been established and constantly reinforced through a variety of resources (as shown in Table 8). The cumulative application of the Goal-Achievement Pattern, together with these micro resources has enhanced the evaluation continuously, which in the end was elevated to a higher level, showing a gradient characteristic (as shown in Fig. 15).

- (2) Phase two: tax cuts for low-income families

**Table 8:** Distribution of evaluation resources in phase one of SPECIFIC one: “tax cuts towards middle class”

Statements	Progressive model	Realization means
Method 1	Establishment	Implicit: IM + graduation resource of SCOPE
Result 1	Cumulative enhancement	Implicit: IM + specific number + graduation resource of EXTENT
Method 2	Establishment	Implicit: IM
Result 2	Cumulative enhancement	Implicit: IM + specific number + infused process of AMOUNT
	Gradient enhancement	Implicit: contrast relationship + “going negativity”

In the second phase, the tax cuts are described as to be beneficial to low-income people which reads as follows:

Phase two of SPECIFIC one: “tax cuts for low-income families”

**Table:** 

Components of semantic pattern	Coded report fragments
Situation 1 & Method 1	(17a) *We eliminated an* < *especially* > ***cruel***[*−*]* tax that fell mostly on Americans making less than $50,000 a year, forcing them to pay ****tremendous****[−] penalties simply because they could not afford government-ordered health plans*[ +]
Method 2 & Result 2	(17b) *We repealed the core of ****disastrous***[*−*] *Obamacare; the individual mandate is now gone. Thank Heavens*[ +]

On the whole, it follows the Goal-Achievement Pattern twice, with < 17a > being the first one and < 17b > being the second. More specifically, < 17a > is composed of Situation and Method, through which a positive evaluation of Trump and his administration is provoked. In Situation, for instance, the tax policy adopted by the previous administration, was condemned for its harshness through explicit expression with high value (“*especially cruel tax*”), which is further enhanced by the coupling of the infused process and the graduation resource of ENHANCEMENT (“*mostly*”) (Hood & Martin, 2007: 390), implying a negative evaluation of the previous administration. The addition of “*simply because*” has further manifested Trump’s strong condemnation of the previous administration, which in turn legalizes the act of “*eliminating the tax*” that Trump and his administration have implemented, revealing once again the function of “going negativity” in realizing evaluative meaning.

In < 17b > , another policy––repealing Obamacare––is presented, which also develops in a Goal-Achievement Pattern, with Method and Result being the constituents. In Method, Trump utilized the explicit attitudinal lexis of “*disastrous*” to openly criticize Obamacare, which has legalized the policy of “*repeal the core of Obamacare*”, thereby invoking a positive evaluation of the present administration. In Result, by stressing that the individual mandate has now disappeared, the benefits of the tax reform are highlighted, thus provoking a positive evaluation of its implementer––the current administration. In addition, the use of “*Thank Heavens*”, which is regarded as dissatisfaction with the policies of the previous administration, as well as a recognition of common religious belief with the audience, amplifies the intensity of evaluation, promoting it to a gradient level.

**Fig. 16:**
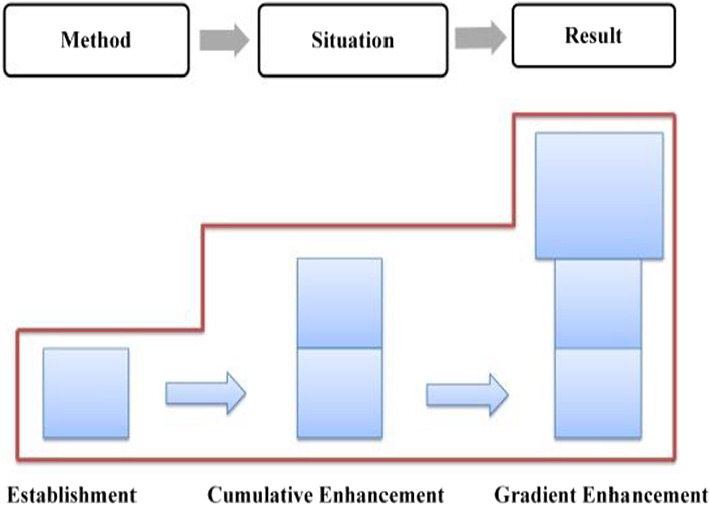
Evaluation feature in tax cuts for low-income families

In summary, along with the report on the tax cut policy for low-income families, a positive evaluation of Trump and his administration is constructed and constantly intensified by both macro means and micro resources. In addition to the two Goal-Achievement Patterns, which provoke evaluative meaning, those micro resources, such as ideational meaning, graduation resources, explicit evaluation lexis, the strategy of “going negativity” and the convergence of religious belief (as shown in Table 9) has cumulatively reinforced the evaluation. Throughout the entire phase, the evaluative meaning developed in a hybrid pattern with both cumulative and gradient enhancements being involved (as shown in Fig. 16).

**Table 9:** Distribution of evaluation resources in phase two of SPECIFIC one: “tax cuts for low-income families”

Statements	Progressive model	Realization means
Method 1	Establishment	Implicit: IM
Sitation 1	Cumulative enhancement	IM: IM + specific number + infused process of AMOUNT + ENHANCEMENT resource
Method 2	Establishment	Implicit: MP
Result 2	Gradient enhancement	Implicit: RP (relational process) + exclamative of sarcasm + going negativity

### Evaluation Feature in Stage Two––“Business Tax Cuts” (SPECIFIC Two)

In SPECIFIC two, Trump stated how tax cuts are beneficial to American businesses and workers (as shown in < 18 >).

SPECIFIC two: business tax cuts

**Table:** 

Components of semantic pattern	Coded report fragments
Method	(18a) *We slashed the business tax rate from 35 percent* < *all the way down* > *to 21 percent, so American companies can compete and win against* < *anyone else* > *,* < *anywhere in the world* > [ +]
Result 1	(18b) *These changes* < *alone* > *are estimated to increase average family income by more than $4,000,* < *a lot of money* > [ +]. *(18c) Small businesses have also received a ****massive***[ +]* tax cut, and can* < *now* > *deduct 20 percent of their business income*[ +]. (18d) Here tonight are Steve Staub and Sandy Kerlinger of Staub Manufacturing––a small business in Ohio. *They have just finished the**** best***[ +]* year in their 20-year history. Because of tax reform, they are handing out raises, hiring an additional 14 people, and expanding into the building next door, ****good***[ +] *feeling*. *One of Staub’s employees, Corey Adams, is also with us tonight. Corey is an all-American worker. He supported himself through high school, lost his job during the 2008 recession, and was later hired by Staub, where he trained to become a welder. Like many hardworking Americans, Corey plans to invest his tax-cut raise into his new home and his two daughters’ education. Corey, please stand, congratulating Corey*[ +]
Result 2	(18e) *Since we passed tax cuts,* < *roughly* > *3 million workers have already gotten tax cut bonuses––* < *many of them* > *,* < *thousands and thousands of dollars* > *per worker, and it’s getting more* < *every month* > *,* < *every week* > [ +]
Result 3	(18f) *Apple has just announced it plans to invest a total of $350 billion in America, and hire another 20,000 workers*[ +]

As a semantic unit, SPECIFIC two develops following the Goal-Achievement Pattern, which is composed of Method and Result, with < 18a > serving as Method, < 18b–18d > as Result one, < 18e > as Result two and < 18f > as Result three.

In Method (as shown in < 18a >), by stating that his administration has drastically reduced the tax rate, a positive evaluative meaning is activated, which is intensified through the coupling of the specific numbers (*35%* vs*. 21%*) and the INTENSITY resource (“*all the way down*”). Moreover, the causal relationship between the clauses has invoked the evaluative meaning of the second clause, which is further intensified by the coupling of the EXTENT resources of “*anyone else*” and “*anywhere in the world*”.

The close connection between the Method and the Results predetermines the evaluation tone of the Results, revealing the radiation function of evaluation. In the first Result (as shown in < 18b–18d >), Trump reports how American people and small businesses have obtained great benefits. Specifically, in < 18b > , by listing the amount of income that American families can obtain from tax reform (“*are estimated to increase average family income by more than $4,000*”), evaluative meaning is afforded, which is further intensified by the coupling of the specific number (“*more than $4,000*”), the EXTENT resource (“*alone*”), and the sub modifying infused expression of AMOUNT (“*a lot of money*”). In < 18c > , by showing how tax cut policy can benefit small businesses, the positive evaluation of Trump and his administration is reinforced. Meanwhile, the alternation of tense from present perfect tense to the simple present tense has euphemistically praise for the current administration, highlighting its credibility. In addition, the explicit attitudinal lexis (“*massive*”) and the verb phrase (“*deduct 20 percent*”) have constantly strengthened Trump’s positive evaluation of the present administration.

Following < 18b > and < 18c > , Trump narrated a real-life story (as shown in < 18d >), which involves how small businesses and ordinary people have obtained benefits from the policy of tax cuts, thereby implying a positive evaluation of the implementer of the policy––Trump and his administration. On the one hand, the radiation function of evaluation gives the narration a positive evaluation; on the other hand, the employment of various evaluative resources at the micro level further reinforces this positive evaluation, in particular, the seemingly objective statement of how a small business named Staub Manufacturing has benefited from the tax reform contains a positive evaluation of the present administration. Meanwhile, the coupling of causal relation (“*because of tax reform*”), explicit attitudinal lexis (“*best*” “*good*”), as well as the graduation resource of EXTENT (“*in their 20-year history*”) has constantly reinforced the evaluation, revealing a prosodic distribution feature of evaluative meaning.

To make the presentation more convincing, Trump mentioned a worker named Corey Adams, who, like many hardworking Americans, lost his job during the 2008 recession but was later hired by Staub, and now plans to invest his tax-cut raise into the new home and his two daughters’ education. The objective statement is companied by the switching of tense, from simple past tense to simple present tense, through which, dissatisfaction with the previous policy, as well as praise for the current policy is expressed, indicating that tense switching in narrative can be used to express evaluative meaning (Fludemik, 1991; Jin, 2009b).

Throughout the first Result, a positive evaluation of the present administration has been accumulating and strengthening.

The second Result, as is shown in < 18e > , presents how the policy of tax cut benefits American people by obtaining tax cut bonuses, which implies a positive evaluation of the current administration. Meanwhile, various quantity phrases (such as “*3 million workers*” “*thousands and thousands of dollars*” “*many of them*” *and* “*even more*”), together with the graduation resources of AMOUNT (“*roughly*” “*every month*” “*every week*”) have made the statement more convincing, thus strengthening the evaluative meaning. In addition, the tense switching happens between the present perfect tense (“*have already gotten*......”) and the present progressive tense (“*and it’s getting......*”) has euphemistically expressed a positive evaluation of the current government by highlighting that American workers have already obtained benefits from tax reform and will continue to get more.

In Result Three, as is shown in < 18f > , by announcing Apple’s investment plan in America, a positive evaluation of the current administration is invoked, which is further intensified by various quantitative phrases, such as “*$350 billion*” and “*another 20,000 workers*”.

**Fig. 17:**
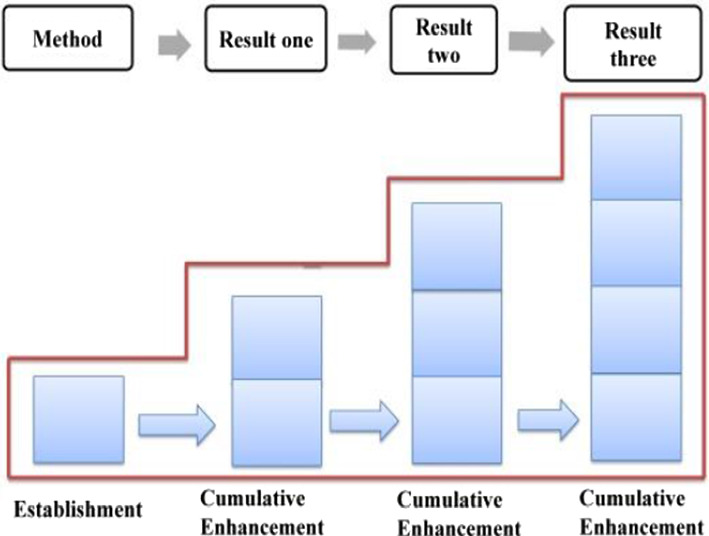
Evaluation feature in business tax cuts

In summary, throughout SPECIFIC two, a positive evaluation of Trump and his administration is established and continuously enhanced. On the one hand, the macro layout of one Method vs. three Results predetermines the characteristics of constant intensification, which means, the evaluative meaning established in the Method will be radiated to the Results, and be constantly reinforced, resulting in a development model of “establishment ^ cumulative enhancement” (as shown in Fig. 17). At the micro level, such resources as ideational meaning, quantity phrases, tense switching, graduation resources, as well as logic-semantic relation between clauses have intercoupled and reinforced the evaluation. The overall distribution of evaluation resources is summarized and listed in Table 10.

**Table 10:** Distribution of evaluation resources in SPECIFIC two: “business tax cuts”

Statement	Progressive model	Realization means
Method	Establishment	Implicit: MP + specific numbers + causal relationship + EXTENT
Result 1	Cumulative enhancement	Implicit: IM + quantity phrases + infused expression + EXTENT + tense switching
Result 2	Cumulative enhancement	Implicit: IM + narration + quantity phrases + + tense switching
Result 3	Cumulative enhancement	Implicit: IM + quantity phrases

## Evaluation Feature in “Macroeconomic Recovery Policy"

The macroeconomic recovery policy is another important issue of Trump’s report, which is as follows:

“Macro-economic recovery policy”

**Table:** 

Components of semantic pattern		Coded report fragments
General		(19a) *In our drive to make Washington accountable, we have eliminated more regulations in our first year than* < *any administration in the history of our country* > [ +]
Specific statement 1	Method 1	(19b) *We have ended the war on American energy, and we have ended the war on beautiful clean coal*[ +]
	Result 1	(19c) *We are* < *now* > < *very* > ***proudly****[* +*]an exporter of energy to the world*[ +]
Specific statement 2	Method 2	(19d) *In Detroit, I halted Government mandates that crippled America’s great beautiful autoworkers, so we can get the Motor City, revving its engines once again, and that’s what’s happening*[ +]
	Result 2	(19e) *Many car companies are now building and expanding plants in the United States, something we have not seen* < *for decades* > [ +]. (19f) *Chrysler is moving a major plant from Mexico to Michigan.* (19 g) *Toyota and Mazda are opening a plant in Alabama, a big one, and we haven’t seen this in a long time, it’s all coming back*[ +]
	Conclusive result	(19 h) < *Very soon* > *, auto plants and other plants will be opening* < *all over our country* > [ +]
Conclusive General		(19i) *This is all news Americans are* < *totally* > *unaccustomed to hearing*[ +]. (19j) < *For many years* > *, companies and jobs were* < *only* > *leaving us. But* < *now* > *they are roaring back, they are coming back, they want to be where the action is, they want to be in the United States of America*[ +]

Judging from the semantic development, it follows the General-Specific Pattern, with < 19a > serving as the General (the Super Theme), < 19b–19c > as Specific 1 (energy policy), < 19d–19 h > as Specific 2 (car industry policy), and < 19i–19j > as the Conclusive Statement (Super New Information).

### Evaluation Feature in the General

In the General (as shown in < 19a >), by stating that the present administration has eliminated more regulations than any administration in the history, a positive evaluation of the present administration is invoked. Meanwhile, the coupling of the graduation resources “*in our first year*” and “*in the history of our country*” has further reinforced the evaluation. In summary, in General, employing semantic contrast, a positive evaluation of Trump and his administration has been released.

### Evaluation Feature in Specific One––“Energy Policy”

Following the General, Trump describes in detail their economic policy from different fields. He begins with the energy policy, which is shown in < 19b > and < 19c > . As a semantic unit, it follows the Goal-Achievement pattern, with < 19b > serving as the Method and < 19c > as the Result.

**Fig. 18:**
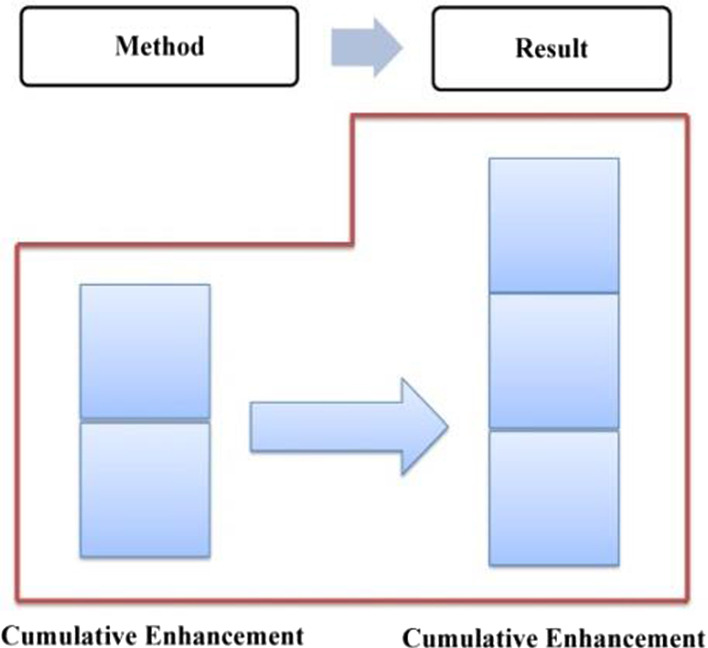
Evaluation feature in energy policy

In Method (as shown in < 19b >), a positive evaluation of the policy implementer––Trump and his administration––is projected through two material processes, which carry ideational meaning and at the same time release the positive evaluative meaning of praising the present administration. In Result (as shown in < 19c >), which is realized by a relational process, the positive evaluation is further enhanced through the affordance of ideational meaning. Meanwhile, the explicit attitudinal lexis of “*proudly*” together with the graduation resources of EXTENT (“*now*” “*very*”) has intensified the evaluation. In addition, tense switching from present perfect tense to simple present tense between < 19b > and < 19c > implicitly echoes the causal relationship, highlighting the effectiveness of the current policy, as well as a positive evaluation of the current administration. In summary, the evaluative meaning in SPECIFIC one is realized by diverse resources (as shown in Table 11), which includes the ideational meaning, causal relationship, tenses switching, attitudinal lexis, as well as graduation resources. Correspondingly, the positive evaluation develops cumulatively (as shown in Fig. 18).

**Table 11:** Distribution of evaluation resources in macro-economic recovery policy: “energy policy” (SPECIFIC one)

Statement	Progressive model	Realization means
Method	Establishment	Implicit: IM
Result	Cumulative enhancement	Implicit: IM + tense switching + causal relationship + attitudinal lexis + EXTENT

### Evaluation Feature in Specific Two––“Automobile Industry Policy”

Following the energy policy, the car industry policy that the current administration has adopted is presented, which is shown from < 19d > to < 19 h > . Semantically, it develops in a Goal-Achievement-Conclusive Achievement Pattern, with < 19d > serving as the Method, < 19e–19 g > as the Result, and < 19 h > as the Conclusive Result.

In < 19d > , Trump pointed out that he has stopped regulations in Detroit that prevented the development of the American auto industry. Behind the ideational meaning, a positive evaluation is invoked, for the attributive clause (“*that crippled America’s great beautiful autoworkers*”) which contains a negative evaluation of the previous administration legalizes the action of “*halted government mandates*”, thus implying a positive evaluation of the present administration. Meanwhile, the use of “*crippled*” not only critiques the inefficacy of previous policies, but also implicitly constructed a political identity for Trump as a therapist, implying a positive evaluation of himself and his administration. In addition, the causal relationships between the clauses, which are accompanied by the tense switching from present perfect to present progressive highlights the excellent working efficiency of the current administration.

In < 19e > , the benefits that the present administration’s automobile industry policy can bring are listed, mainly by stating that the United States is attracting many car companies to build or expand plants. The statement itself has afforded praise for Trump and his administration, within which, the appositive structure (“*something we have not seen for decades*”) has further intensified the evaluation by providing more specific information. Following < 19e > , car brands (Chrysler, Toyota, and Mazda) that are opening plants in America(< 19f >–< 19 g >) were listed, with more graduation resources of EXTENT being added, such as “*from Mexico to Michigan*” and “*in Alabama*”. As a result, the positive evaluation sounds more well-founded and persuasive. Moreover, tense switching from present progressive to present perfect and then to present progressive once again implicitly reveals dissatisfaction with the previous administration and admiration for the current one.

In Conclusive Result, as is shown in < 19 h > , the positive evaluation of the present administration is further reinforced through the ideational meaning itself, which promises a bright future for the American auto industry and other industries by stating that the plants will be opening all over the country. This implies commendation for the governing capacity of the present administration.

**Fig. 19:**
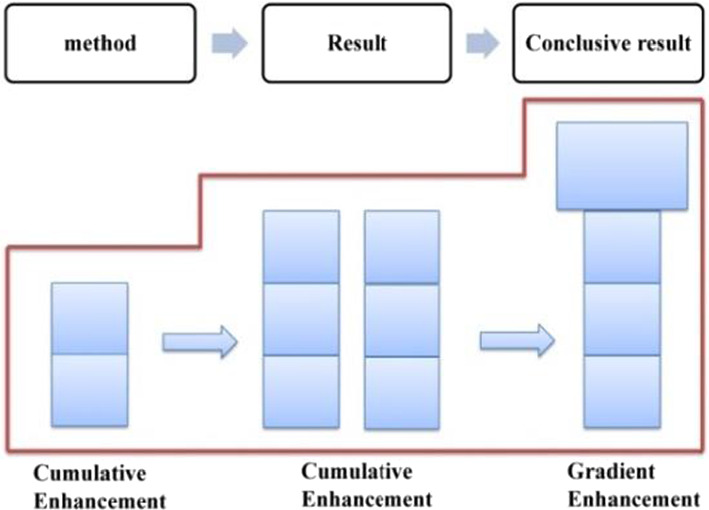
Evaluation feature in automobile industry policy

Throughout SPECIFIC two, the semantic pattern of Method ^ Result ^ Conclusive Result presupposes, from a macro perspective, the radiation and sustained intensification characteristics of evaluative meaning. On the micro level, ideational meaning, causal relation, narration, tense switching, as well as various graduation resources have intercoupled and sustainedly reinforced the evaluative meaning (as shown in Table 12). In general, the evaluative meaning is established and constantly enhanced, evolving from cumulative to gradient, which is mainly determined by the Pattern of Goal-Achievement-Conclusive Achievement (as shown in Fig. 19).

**Table 12:** Distribution of evaluation resources in macro-economic recovery policy: “automobile industry policy” (SPECIFIC two)

Statement	Progressive model	Realization means
Method	Establishment	Implicit: IM + causal relation + conceptual metaphor
Result	Cumulative enhancement	Implicit: IM + appositive structure + narration
Conclusive result	Gradient enhancement	Implicit: IM + tense switching + EXTENT
Conclusive general	Cumulative enhancement	Implicit: IM + tense switching + repetitive expressions

### Evaluation feature in the Conclusive General

In Conclusive General, as is shown in < 19i–19j > , Trump begins with a summary, saying that American people are totally uncustomed to hearing the news. In the following, “going negativity” is deployed to stress how poor previous policies are, which in effect implies a positive judgment of the current administration’s policy. Besides this, within < 19j > , the tense switching from past continuous tense to present progressive tense has highlighted, to the audience, the high efficiency of the current administration, which is further intensified by the repetitive expressions (“*they are roaring back, they are coming back*”).

Throughout the entire issue of “macro-economic recovery policy”, a positive evaluation of Trump and his administration has been established and continuously reinforced through the intercoupling of various resources, both explicit and implicit, lexical and syntactic, as well as intra-clause and inter-clause.

## Evaluation Feature in “Trade Deals Issue”

The last economic issue involves the policy of American international trade, which is shown in the following.

“Trade deals issue”

**Table:** 

Components of semantic pattern	Coded report fragments
Problem	(20a) *America has also finally turned the page on decades of ****unfair***[−] *trade deals that sacrificed our prosperity and shipped away our companies, our jobs, and our wealth*[ +]. (20b) *Our nation has lost its wealth*[−]*, but we are getting it back* < *so* > ***fast***[ +][ +]. (20c) *The era of economic ****surrender****[−] is* < *totally* > *over*[ +]
Approach	(20d) < From now on > , we expect trading relationships to be **fair**[ +], and < very importantly > , **reciprocal**[ +]. (20e) We will work to fix **bad**[−] trade deals and negotiate **new**[ +] ones. (20f) And they will be **good**[ +] ones and they will be **fair**[ +]. (20 g) *And we will protect American workers and American intellectual property, through strong enforcement of our trade rules*[ +]

In general, it follows the Problem-Approach Pattern, with the Problem including < 20a–20c > , and the Approach including < 20d–20 g > . In < 20a > , the stark difference between the economy state under the previous administrations and the current one is highlighted through “going negativity”, with the previous trade deal being portrayed as “*unfair*” “*sacrificed our prosperity and shipped away our companies, our jobs, and our wealth*”. This in effect releases a positive evaluation of the current administration, thus legalizing and naturalizing its policy of negotiating new trade deals. In < 20b > and < 20c > , the adversative relation between clauses invokes a positive evaluation of the current administration, which is reinforced by the tense switching between present perfect tense to simple present tense. Meanwhile, the graduation resources of EXTENT (“*so*” and “*totally*”), which are utilized to modify the explicit attitudinal lexis (“*fast*” and “*surrender*”), further strengthened the intensity of evaluation.

Starting from < 20d > , the specific plans that the current administration will adopt in the next year are stated. To be more specific, the coupling of ideational meaning and explicit attitudinal lexis (“*fair*” “*reciprocal*”) in < 20d > invokes praise for Trump and his administration since their trade policy is described as conforming to the interest of the American people. In the following, by describing the previous administration’s trade deals as “*the bad ones*”, while the present as “*new and good ones*” (as shown in < 20e >–< 20f >), the positive evaluation is further strengthened. In < 20 g > , by stating that the current administration will “*protect*” American workers and American intellectual property through enforcing a more reciprocal trade deal, a positive evaluation is also afforded.

**Fig. 20:**
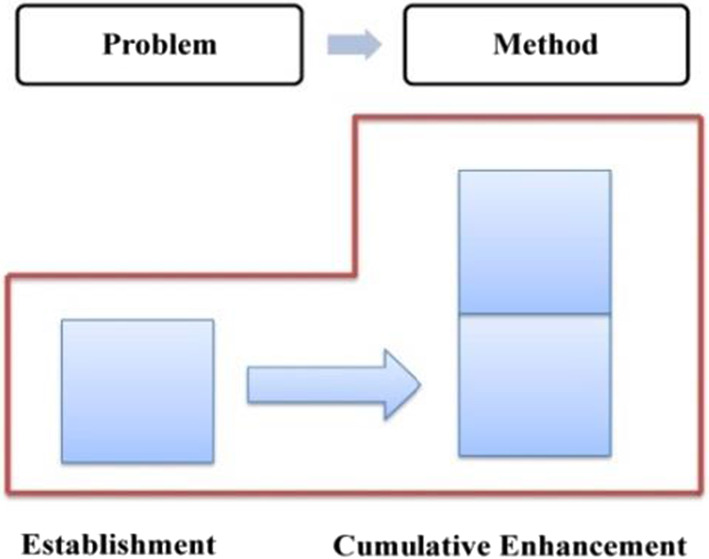
Evaluation feature in trade deals issue

Throughout the entire “trade deals issue”, tense switching happens frequently among present perfect, present progressive, simple present tense and simple future tense, which, accompanying the ideational meaning, implicitly delivers evaluative meaning. In addition to this, other evaluation resources such as explicit attitudinal lexis, various graduation resources, and semantic relations across clauses have intensified this positive evaluation to varied degrees, resulting in a cumulative characteristic, which can be shown in Fig. 20.

## Trump’s Evaluation Mechanism in SUA

At the discourse semantic level, evaluative meaning is produced and reinforced by the “joint efforts” of all language systems. As demonstrated by the above analysis, a range of linguistic resources are employed to express evaluative meaning, leading to diverse evaluation mechanisms.

- (1) Coupling mechanism

As one of the most prominent evaluative mechanisms (Knight, 2010; Martin, 2010; Stenglin, 2004), coupling refers to the combination patterns of choices made from a meaning potential (Martin, 2008: 491), which can be composed of two, three, four, o more choices. Coupling can be both synchronically combined, for instance, “*very sad*” exemplifies the combination of attitudinal and graduation resources, and diachronically combined, for example, the relationship between “*very sad*” and “*extremely unhappy*”. To enhance the evaluative meanings, Trump employs a variety of coupling mechanisms, among which the coupling of attitudinal and graduation resources is rather common. By coupling, the subjective evaluation is sustainedly reinforced, thereby guiding the audience to make a similar evaluation.

- (2) Semantic prosody mechanism

In addition to coupling, the mechanism of semantic prosody is also widely used, revealing the prosodic realization of evaluative meaning (Martin & White, 2005). There are different types of semantic prosody, such as saturation, intensification, domination (Martin & White, 2005: 18–23), wave prosody, radiation prosody and gradient prosody (Dong & Li, 2021). Regarding the present study, Trump prefers intensified prosody and radiation prosody, which cater to the cumulative reinforcement characteristics of evaluative meaning. The mechanism of semantic prosody aligns with the fundamental characteristics of text/discourse. As a semantic unit, text/discourse is essentially coherent. Evaluative meaning is typically realized by various lexical or grammatical resources within clauses. However, the semantic coherence of a discourse/text determines that the evaluative meaning occurring within clauses permeates the surrounding sentences or utterances, resulting in the prosodic realization and continuous intensification of evaluative meaning.

- (3) Tense switching mechanism

Tense switching is the third mechanism that permeates the entire report, which serves as a subtle but important evaluative device. As a basic grammatical category, tense works with verbs, as well as other means to construct temporal connections between situations. However, tense is not solely a category that realizes ideational meaning, but also serves to reveal interpersonal meaning (Palmer, 1989; Thompson, 1996: 58). In a complete text or discourse, tenses always alternate (Li, 2002). Tense switching can realize evaluative meaning (Fleischman, 1990: 145), through which, the speaker or writer quietly infiltrates his into the so-called “neutral” statements. As far as this study is concerned, Trump has frequently employed the mechanism of tense switching to simultaneously convey a negative evaluation of the previous administration and a positive evaluation of his administration, among which the most common one occurs between the simple past tense and present progressive, as well as the present perfect and present progressive, with the first tense of each pair being used to implicitly criticize the previous policy, while the second to praise the current administration led by himself.

## Conclusion

**Fig. 21:**
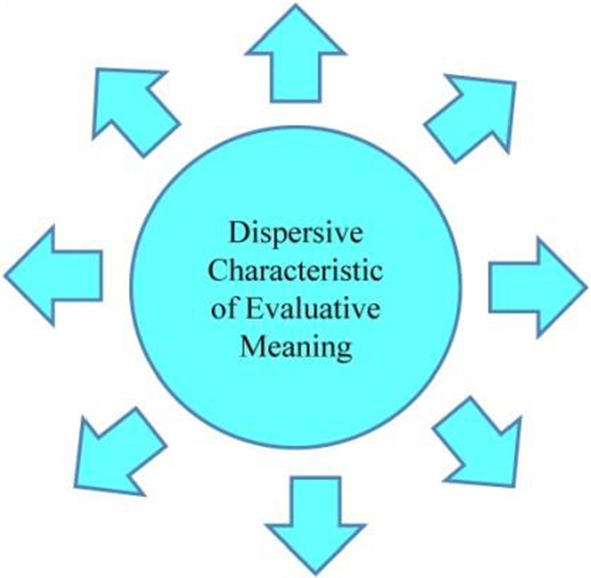
Dispersive characteristic of evaluative meaning

Taking the economic issue of Trump’s first SUA as the original data, this study aims to explore the evaluation features of political speeches, with both micro and macro dimensions being taken into consideration. On the macro level, various semantic patterns have been found, with Goal-Achievement Pattern and General-Example being the most common ones, which predetermine the evaluative tone, giving the following statements evaluative meanings, revealing the radiating nature of evaluative meaning at the discourse semantic level (as shown in Fig. 21). At the micro level, a variety of resources have been identified, both explicit and implicit, lexical and syntactical, attitudinal and gradational, which collaborate to reinforce the subjective evaluation, revealing the holistic characteristic in the realization of evaluative meaning.

Throughout the study, three evaluative mechanisms have been proposed, which are the coupling of meaning, semantic prosody, and tense switching. They collaborate, promoting the subjective evaluation to be established and reinforced in a cumulative, gradient or hybrid pattern.

The present study has not only revealed Trump’s political discourse feature, to some extent, but also demonstrated the applicability of a holistic research paradigm in political discourse analysis, which has illuminating implications for promoting the qualitative research of evaluation. Critical discourse analysis plays a role in revealing secrets (Zhang and Akhtar, 2021). Future studies can integrate qualitative research with critical discourse analysis to figure out the underlying social or psychological mechanisms, thus facilitating a more accurate interpretation of political discourse.

## Data Availability

The original contributions presented in the study are included in the article/supplementary material, further inquiries can be directed to the corresponding author.
